# A Shallow U-Net Architecture for Reliably Predicting Blood Pressure (BP) from Photoplethysmogram (PPG) and Electrocardiogram (ECG) Signals

**DOI:** 10.3390/s22030919

**Published:** 2022-01-25

**Authors:** Sakib Mahmud, Nabil Ibtehaz, Amith Khandakar, Anas M. Tahir, Tawsifur Rahman, Khandaker Reajul Islam, Md Shafayet Hossain, M. Sohel Rahman, Farayi Musharavati, Mohamed Arselene Ayari, Mohammad Tariqul Islam, Muhammad E. H. Chowdhury

**Affiliations:** 1 Department of Electrical Engineering, Qatar University, Doha P.O. Box 2713, Qatar; sakib.mahmud@qu.edu.qa (S.M.); 1017052037@grad.cse.buet.ac.bd (N.I.); amitk@qu.edu.qa (A.K.); a.tahir@qu.edu.qa (A.M.T.); tawsifur.rahman@qu.edu.qa (T.R.); k.reajul@qu.edu.qa (K.R.I.); 2 Department of Electrical, Electronics and Systems Engineering, Universiti Kebangsaan Malaysia, Bangi 43600, Selangor, Malaysia; p108100@siswa.ukm.edu.my (M.S.H.); tariqul@ukm.edu.my (M.T.I.); 3 Department of CSE, BUET, ECE Building, West Palashi, Dhaka 1205, Bangladesh; msrahman@cse.buet.ac.bd; 4 Department Mechanical and Industrial Engineering, Qatar University, Doha P.O. Box 2713, Qatar; farayi@qu.edu.qa; 5 Department of Civil and Architectural Engineering, Qatar University, Doha P.O. Box 2713, Qatar; arslana@qu.edu.qa; 6 Technology Innovation and Engineering Education (TIEE), Qatar University, Doha P.O. Box 2713, Qatar

**Keywords:** systolic blood pressure, diastolic blood pressure, arterial blood pressure, photoplethysmogram, electrocardiogram, autoencoder, feature extraction

## Abstract

Cardiovascular diseases are the most common causes of death around the world. To detect and treat heart-related diseases, continuous blood pressure (BP) monitoring along with many other parameters are required. Several invasive and non-invasive methods have been developed for this purpose. Most existing methods used in hospitals for continuous monitoring of BP are invasive. On the contrary, cuff-based BP monitoring methods, which can predict systolic blood pressure (SBP) and diastolic blood pressure (DBP), cannot be used for continuous monitoring. Several studies attempted to predict BP from non-invasively collectible signals such as photoplethysmograms (PPG) and electrocardiograms (ECG), which can be used for continuous monitoring. In this study, we explored the applicability of autoencoders in predicting BP from PPG and ECG signals. The investigation was carried out on 12,000 instances of 942 patients of the MIMIC-II dataset, and it was found that a very shallow, one-dimensional autoencoder can extract the relevant features to predict the SBP and DBP with state-of-the-art performance on a very large dataset. An independent test set from a portion of the MIMIC-II dataset provided a mean absolute error (MAE) of 2.333 and 0.713 for SBP and DBP, respectively. On an external dataset of 40 subjects, the model trained on the MIMIC-II dataset provided an MAE of 2.728 and 1.166 for SBP and DBP, respectively. For both the cases, the results met British Hypertension Society (BHS) Grade A and surpassed the studies from the current literature.

## 1. Introduction

Despite tremendous advancements in the healthcare sector, cardiovascular diseases (CVDs) still secured the top positions last year in the list of leading causes of death globally. The most fatal CVD was Ischaemic Heart Disease which is termed by the World Health Organization (WHO) as the “world’s biggest killer” as it accounted for 16% of the total deaths from 2000 to 2019 [1]. The second, third, and fourth positions were secured by stroke, chronic pulmonary diseases and lower respiratory infections, respectively which are also, directly and indirectly, related to CVDs [2,3,4]. Hypertension or high blood pressure (BP) is one of the leading causes of CVDs: Almost 54% of strokes and 47% of coronary heart diseases, worldwide, can be attributed to high BP [5]. In the USA alone, there are around 67 million people (almost one-third of the population) suffering from various hypertension problems while the irony is, according to this statistic [6], more than half of them are reluctant to mitigate their condition. The main reason behind this kind of reluctance seen among high BP patients is the dormant nature of hypertension which eventually leads to untimely death. For this reason, it is commonly termed the “Silent Killer” [7]. Due to the silent nature of hypertension, it is crucial to continuously monitor the BP of the patients. Due to a shortage of expert physicians compared to the huge number of patients, automated BP monitoring methods seem to be a viable alternative in this regard.

Several studies attempted to tackle this problem following various methodologies and a handful of them (e.g., [8]) were adopted by the healthcare centers to measure blood pressure continuously. However, most of the robust methods for blood pressure monitoring are either intermittent or invasive if continuous. Two commonly used BP recording methods are the cuff-based oscillometric technique and arterial blood pressure (ABP) reading from the radial artery through cannulation. Both of these methods are reliable; the former is non-invasive albeit intermittent whereas the latter is continuous but invasive [9]. A few calibration-based techniques have been developed to detect BP non-invasively, such as the photoplethysmography (PPG)-based finger-clamp method [10] and the applanation tonometry method [11]. Calibration is required for both of these non-invasive techniques since they do not readily provide the correct BP values or ABP signals. This type of calibration or mapping can also be useful in measuring BP readings [12] or ABP waveforms [13] from PPG if they are recorded simultaneously. Recently, a few studies have been reported where various machine learning (ML) and statistical techniques are used to predict BP from non-invasively collected PPG signals. Some studies used traditional ML (regression) models, such as support vector regressor (SVR) [14], adaptive boosting (AdaBoost) [12], random forest [15], gradient boosting (GradBoost) [16], Gaussian process regression (GPR) [17], artificial neural network (ANN) [18,19,20,21], recurrent neural network (RNN)-based long short-term memory (LSTM) [22,23], etc. on small or medium-sized datasets to predict BP from PPG alone or a combination of PPG and electrocardiogram (ECG). 

In recent years, convolution neural network (CNN)-based deep learning (DL) techniques have been utilized to solve complex problems on large datasets in 1D (e.g., Signals), 2D (e.g., images, and even in 3D (e.g., videos) settings. However, there are not many deep learning-based approaches in the literature for BP estimation. Slapnicặr et al. [24] predicted BP from PPG, its derivatives (1D signals) and their respective spectrograms (2D signals) using a hybrid pipeline containing both 1D and 2D CNNs termed as “Spectro-Temporal ResNets”. Recently, Athaya et al. [25] used modified U-Net [26] architecture for PPG to ABP signal to signal translation. On the other hand, Ibtehaz et al. [13] in their work used two CNN networks in sequence (U-Net and MultiResUNet [27]) for PPG to ABP signal translation but could not reach Grade A [28] for systolic blood pressure (SBP) prediction. Therefore, based on the current literature, there is still scope for significant improvement in BP predictions using deep learning models.

U-Net is an encoder-decoder-based deep CNN architecture that was originally used for image (2D) segmentation. Many studies used U-Net to perform tasks, such as biomedical image segmentation [29], shape regeneration [30], road shape extraction from satellite maps [31], etc. A good number of studies tried to design special-purpose variations of U-Net such as U-Net++ [29], nnu-Net [32], Ternausnet [33], Wave-U-Net [34], etc. for various applications, mostly in 2D settings. There is also a 3D version of U-Net [35] designed for tackling three-dimensional problems. In the 1D domain, apart from the PPG to ABP signal translation discussed earlier, there have also been works in speech enhancement [36], echo cancellation [37], heartbeat detection, etc. Thus, the U-Net architecture has been modified in various ways for solving different types of problems and in a few cases, a shallow U-Net performed better than a deeper version. For example, Wu et al. [38,39] utilized a shallow three-layer version of U-Net used for shadow detection as part of a scene understanding task. On the other hand, to the best of our knowledge, the U-Net architecture has rarely been used just for feature extraction while acting as an autoencoder. These studies [37,40,41,42] tried to extract features from PPG and/or ECG signals using generic CNNs and used those features on LSTM models to predict BP. Features were extracted separately from PPG and ECG and both were put into LSTM networks to separately predict SBP and diastolic blood pressure (DBP). In this study, we followed a similar approach but for feature extraction, we utilized the encoder portion of the U-Net. A densely connected multi-layer perceptron (MLP) layer was added to the end of the encoder for extracting the network learned features. This lightweight version of the U-Net can easily be applied to devices in a (computing and memory) resource-constrained setting. Thus, the novelty of this work lies not only in the feature extraction pipeline but also in using the shallowest version of U-Net on a large dataset for extracting features optimizing the BP prediction process. To the best of our knowledge, our extracted latent features from the shallowest U-Net have outperformed most of the BP prediction techniques found in the literature so far.

## 2. Materials and Methods

### 2.1. Datasets

In this study, two different datasets have been used, which are briefly described below.

#### 2.1.1. Multi-Parameter Intelligent Monitoring in Intensive Care II (MIMIC-II) Dataset from the UCI Repository

The Cuff-Less Blood Pressure Estimation Dataset [14] from the UCI Machine Learning Repository [43], termed as the “UCI Dataset”, has been used in this study. The UCI Dataset is a filtered and processed version of the Multi-Parameter Intelligent Monitoring in Intensive Care II (MIMIC-II) Waveform database [44,45]. The MIMIC-II Waveform database contains records of continuous high-resolution physiologic waveforms and minute-by-minute numeric trends of physiologic measurements, such as ABP, PPG, cerebral perfusion pressure (CPP), central venous pressure (CVP), pulmonary arterial pressure (PAP), so on and so forth. The UCI Dataset contains 12,000 instances of simultaneous PPG, ABP, and ECG data of 942 patients extracted from the MIMIC-II Waveform database with a sampling rate of 125 Hz. The 12,000 instances of the UCI Dataset were uniformly divided into four parts, each part containing 3000 instances, and the data are available in MATLAB file format (“.mat”). Even though the MIMIC-II database has data from a large number of patients, only 942 patients had all three PPG, ECG, and ABP signals simultaneously, which is required for BP prediction in the proposed model. UCI Dataset was created with only the MIMIC-II records where all three of PPG, ABP, and ECG data were present. While creating the UCI Dataset, Kachuee et al. [14] performed some signal processing tasks, such as smoothing all signals using a simple averaging filter, removing signals with unacceptable human BP and heart rate (HR) values, getting rid of signals with severe discontinuities and auto-correlating PPG signals for checking the similarity between successive pulses. Therefore, these steps were not repeated in this study.

#### 2.1.2. Ballistocardiogram (BCG) Dataset

The external validation dataset used in this work has been collected and shared recently by Carlson et al. [46] (referred to as “BCG Dataset” in this paper). Several heart-driven signals, such as ballistocardiogram (BCG), ECG, PPG, and ABP waveforms are available in the dataset. Note that BCG waveforms of this dataset are not of any interest for this study. Data were collected from 40 subjects (17 males and 23 females) with a sampling rate of 1000 Hz. The signals were digitized by the NI-9220 [47] device, which was used to gather signals collected by various data acquisition devices. The ABP signals in this dataset were non-invasively collected from the reconstructed brachial artery pressure (reBAP) signals, which were collected using Finometer Pro [48] from Finapres Medical Systems. The ABP signals were represented in terms of volts following a normalizing scale of 100 mmHg/volt. The BCG Dataset is also available in the “.mat” file format. An overview of both datasets is provided in Table 1.

As seen from Table 1, the UCI dataset, even though larger, is more deviated, especially for SBP. On the other hand, DBP and mean arterial pressure (MAP) of the signals in the UCI dataset vary within a much wider range than those in the BCG dataset. The sampling rate of the signals in the BCG dataset was resampled at 125 Hz from 1000 Hz to maintain harmony with the UCI dataset signals. So, the duration of a sample signal was about (1024/125) ≈ 8.192 s. This means that the total duration of the data collected from the UCI dataset was about 456 h and was around 4.26 h for the BCG dataset.

### 2.2. Data Pre-Processing

**Figure 1:**
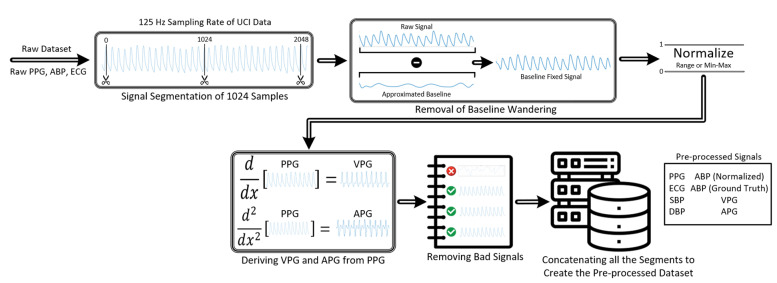
Flowchart representing the data pre-processing pipeline for the UCI Dataset. The pipeline for the Ballistocardiogram (BCG) Dataset is almost identical except the ABP signals were denormalized first by multiplying with the normalizing factor of 100.

At first, the signal was segmented to 1024 samples from the UCI dataset while preserving the original sampling rate of 125 Hz. Signals from the UCI dataset suffer from severe baseline drift in many instances. Therefore, baseline wandering was removed before normalizing the signals. After fixing the baseline drifts and properly normalizing the signals, the first two derivatives of PPG were derived and stored along with their corresponding PPG signals to be used as predictors alongside PPG and ECG. Before compiling the whole dataset, highly distorted signals were removed. Signal pre-processing was performed in MATLAB (version R2020a). The whole data pre-processing procedure is shown in Figure 1. The BCG dataset was also pre-processed similarly. However, before pre-processing, their sampling frequency was down-sampled from 1000 Hz to 125 Hz to ensure consistency with the UCI dataset. The ABP signals in the BCG dataset were denormalized by multiplying with a factor of 100 since they were normalized and stored by maintaining a scale of 100 mmHg/volt. To better understand the algorithms used for data pre-processing, pseudo-codes were used to explain each process in detail. The MATLAB built-in functions used in the code have been written in italics in the pseudo-codes.

Baseline drift correction: this was undertaken using the built-in functions of MATLAB (‘movmin’ [49], ‘polyfit’ [50], and ‘polyval’ [51]). At first ‘movmin’ or moving minimum function was used to find an array of estimated minimum points acting as a baseline approximation for the waveform. Afterward, the ‘polyfit’ function was used to fit a higher-order polynomial along with the estimated points and ‘polyval’ was used to formulate the polynomial based on the ‘polyfit’ result, which is the estimated baseline. Then, the baseline was deducted from the raw signal to achieve the baseline drift corrected signal. The Algorithm 1 pseudo-code for baseline drift correction is shown below.
**Algorithm 1** Pseudo-Code: Baseline Drift Correction**Inputs:** X (Segmented Raw Signal)1.1.1. **Check** X is a Row Vector **else** X = Transpose (X)1.2.1. **Initialize** Time_Vector = Transpose (*linspace*(1, *length*(X), *length*(X)))2.1.1. **try:**2.2.1.    [peaks, peak_locations] = *findpeaks*(X)2.3.1.    **Initialize** peak_dist2.4.1.    **for** i = 1: (*length*(peak_locations) − 1)2.4.2.      peak_dist(i) = peak_locations(i + 1) − peak_locations(i)2.4.3.    **end for**2.5.1.    median_peak_dist = *median* (peak_dist)2.6.1.    Baseline = *movmin* (X, median_peak_dist)2.7.1.    P = *polyfit* (Time_Vector’, Baseline, *round*(median_peak_dist))3.1.1. **except:**3.2.1.    **Initialize** polynomial_order3.3.1.    P = *polyfit* (Time_Vector’, X, polynomial_order)4.1.1. Baseline_Fit = *polyval* (P, Time_Vector’)4.2.1. Y = X – Baseline_Fit4.3.1. Y = Y – *min*(Y)4.4.1. X_amp = *max*(X) − *min*(X)4.5.1. Y_amp = *max*(Y) − *min*(Y)4.6.1. Y = Y*(X_amp/Y_amp)**Outputs:** Y (Baseline Corrected Signal)

Normalization: PPG and ECG signals were z-score normalized, followed by a range normalized between 0 and 1 per segment (Equation (1)) while ABP waveforms were min-max normalized globally, in terms of the minimum and maximum of the ABP waveforms across the whole dataset (Equation (2)).

(1) $$PPG_{i}\left(norm_{PPG}\right)=range\left(\left(\frac{PPG_{i}-\mu_{i}}{\sigma_{i}}\right),\left[0\;1\right]\right)$$

ABP signals were not range normalized between 0 to 1 to retain their relative amplitude feature (i.e., BP levels) which was found to be helpful during BP prediction. Mentionable that the bold quantities in the equations mean signals, similar to vectors.

(2) $$ABP_{i}\left(norm_{BP}\right)=\frac{ABP_{i}}{SBP_{Global\;Maximum}}$$

**Figure 2:**
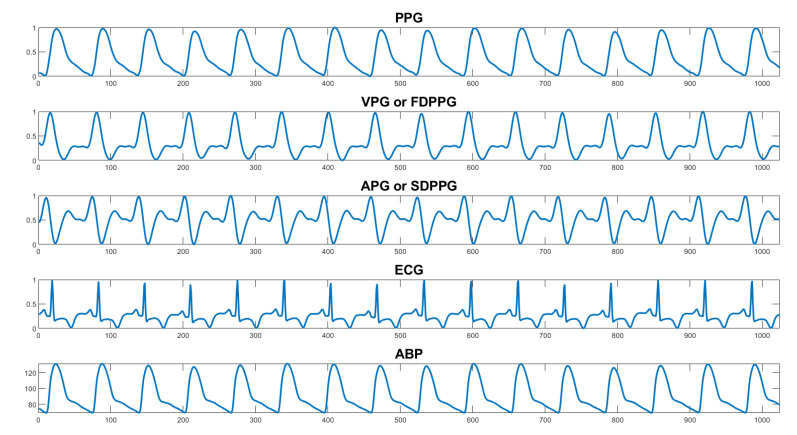
Snapshot of five signals (photoplethysmography (PPG), velocity of PPG (VPG), acceleration of PPG (APG), electrocardiogram (ECG), and arterial blood pressure (ABP)) from a segment of the UCI dataset after pre-processing.

Derivatives of PPG: according to literature, the first and second derivatives of PPG also provide valuable information or features while predicting BP. They are called various names such as PPG’, PPG’’ or Velocity of PPG (VPG), Acceleration of PPG (APG), or FDPPG (First Derivative of PPG), SDPPG (Second Derivative of PPG) [52,53,54] (Figure 2). To find the VPG and APG from PPG, MATLAB’s ‘diff’ function was used. However, a finite “Step Size” [55] of the “diff” function induced distortions in the derived signals which kept increasing for higher-order derivatives. To remove these high-frequency distortions, the signals need to be filtered in each stage, which was done using MATLAB’s “designfilt” function [56,57]. The cutoff frequencies for the bandpass filter were set carefully to pass through important frequency components related to PPG derivatives while attenuating low and high-frequency distortions. However, applying a filter on the signals creates some delay which deteriorates along with the derivative order (APG > VPG). MATLAB’s built-in function ‘grpdelay’ [58] was used to find the average filter delay. Then the signals were moved to the left by the amount of their respective delay. Adjustment of the length of original PPG signals was undertaken to ensure the length of VPG and APG signals after the delay to maintain the length of the signals at 1024. The Algorithm 2 pseudo-code for deriving PPG derivatives is shown below.
**Algorithm 2** Pseudo-Code: Deriving PPG Derivatives**Inputs:** PPG1.1.1. **Initialize** Bandpass Filter Parameters (Filter Order, Passband, Stopband, Sampling Frequencies)2.1.1 bandpass_filter = *designfilt*(Bandpass Filter using Filter Parameters)2.2.1 delay = *mean*(*grpdelay*(bandpass_filter))3.1.1 **Initialize** Sample_Num = *linspace*(1, *length*(PPG), *length*(PPG))3.2.1 dt = Sample_Num(2) − Sample_Num(1) 3.3.1 PPG = **Normalize**(PPG) 3.4.1 VPG **= Normalize**(bandpass_filter(PPG)/dt) 3.5.1 APG **= Normalize**(bandpass_filter(VPG)/dt) 3.6.1 PPG = PPG(1:end−2*delay) 3.7.1 VPG = VPG(delay+1:end) 3.8.1 APG = APG(2*delay+1:end) **Outputs:** PPG, VPG, APG

Removing bad signals: the signal samples extracted from the UCI dataset contains many highly distorted signals that can potentially affect the performance of the deep learning model significantly as the network tries to learn from them. Hence, the following types of samples were removed from the dataset: ABP signals with extreme SBP and DBP values, blank samples, and signals which exceed a certain distortion threshold. In particular, ABP signals with SBP values smaller than 80 and greater than 190, DBP values greater than 120 and smaller than 50, and ABP signals which had a BP range (SBP–DBP) less than 20 or more than 120 were removed since it was observed that apart from some extreme cases, highly distorted signals normally had such a BP range. Under this scheme, around 2% of signals were removed from the datasets. After performing some signal processing and taking the derivatives, a few samples became blank due to being extremely distorted; these were also removed. There are levels of distortions for various samples and a sample remains acceptable up to a certain level of distortion. As shown in Supplementary Figure S1, for ABP and PPG signals, the distorted samples had two main traits, namely highly non-uniform peaks either in terms of distance or height, and double peaks. Standard deviation (STD) of the peak-to-peak distances and peak prominences (relative height) were observed to detect this anomaly and signals were sorted out based on a threshold of deviation. This threshold was set after performing trial and error by manually observing more than 1000 samples. The Algorithm 3 pseudo-code for removing bad signals is shown below.
**Algorithm 3** Pseudo-Code: Deriving PPG Derivatives**Inputs:** PPG, ABP, Signal_Length 1.1.1 Normalize both signals 1.2.1 PPG_Size = *size*(PPG) 1.3.1 ABP_Size = *size*(ABP) 1.4.1 **if** (PPG_Size(1) or ABP_Size(1)) > 1 **then** Transpose 2.1.1 **Initialize** Time_Vector = *linspace*(1, *length*(PPG), *length*(PPG)) 2.2.1 [peaks_PPG, peak_locations_PPG] = *findpeaks*(PPG) 2.3.1 num_peaks_PPG = *length*(peaks_PPG) 2.4.1 std_peaks_PPG = *std*(peaks_PPG) 2.5.1 std_peaks_dist_PPG = *std*(peak_locations_PPG) 2.6.1 [peaks_ABP, peak_locations_ABP] = *findpeaks*(ABP) 2.7.1 num_peaks_ABP = *length*(peaks_ABP) 2.8.1 std_peaks_ABP = *std*(peaks_ABP) 2.9.1 std_peaks_dist_ABP = *std*(peak_locations_ABP) 3.1.1 **Initialize** thresholds 3.2.1 **if** (std_peaks_PPG, std_peaks_dist_PPG, std_peaks_ABP, std_peaks_dist_ABP, num_peaks_PPG, num_peaks_ABP) satisfies thresholds **then** Decision = 0 3.2.2 **else** Decision = 1 **Outputs:** Decision (0 or 1)

**Figure 3:**
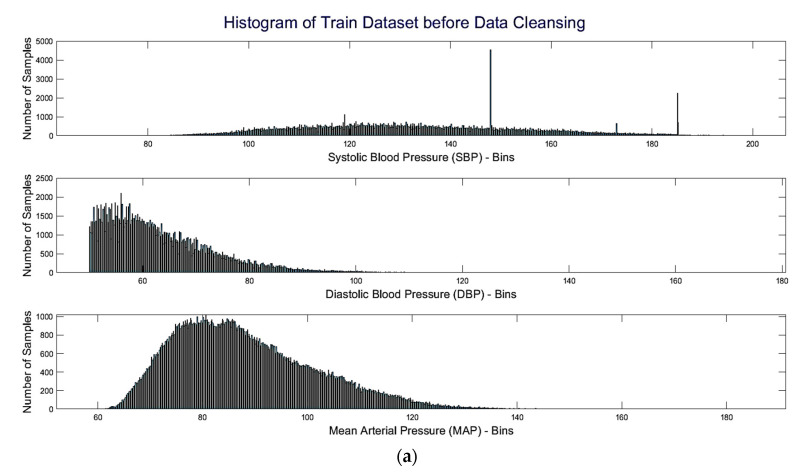

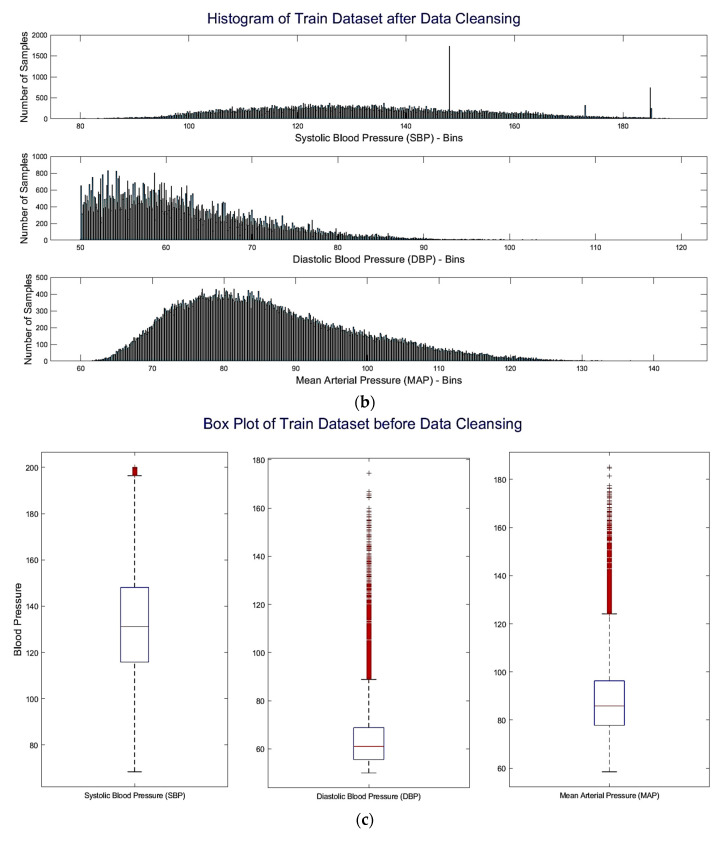

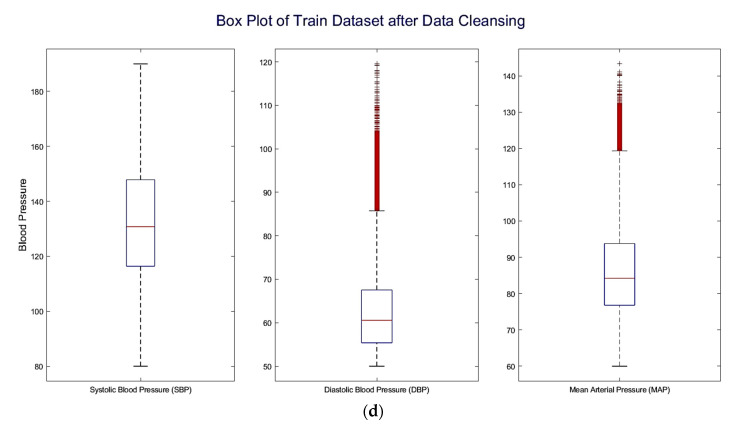
Histogram (**a**,**b**) and box plot (**c**,**d**) of systolic blood pressure (SBP), diastolic blood pressure (DBP), and mean arterial pressure (MAP) of signals in the training set before (**a**,**c**) and after (**b**,**d**) data cleansing, respectively.

Histograms of ABP and SBP in Figure 3a,c can be compared for the signal distribution before and after the signal pre-processing. The box plots in Figure 3 show that after removing the low-quality signals, the number of outliers decreased, and a greater portion of signals entered into the interquartile range. Removing these outliers might improve the performance of the network. The median and standard deviation have changed marginally as the signal distribution is spreading more. Around 25% of both train and test signals were removed through this “bad signal removal” scheme. It is worth mentioning that most other researchers also worked on ABP signals of a certain BP range alongside putting on other constraints to boost the network performance [13,22,23,24,25]. Even though a considerable number of segments were removed, due to the use of more than one channel and considering the whole UCI version of the MIMIC-II dataset, a comparatively larger number of segments were available for training, validation, and testing.

### 2.3. Rationale behind This Study

The rationale behind this study was to extract an effective set of features from a very large dataset containing PPG, ECG, and ABP signals which can be used to reliably predict BP. While studies mostly use PPG and ECG signals for BP prediction or extracting features directly, we propose to use an approach inspired by the power of autoencoders to extract the latent features automatically and check for the performance. In traditional autoencoders, usually, the input is given to the network to reconstruct it through a latent space compact transformation. This enforces the model to learn the distinctive attributes of the input and thus has shown great success in feature extraction. Therefore, an obvious idea would be to train an autoencoder using PPG and ECG signals both as inputs and outputs. This will provide us with a latent space aware of the diverse patterns of the PPG and ECG signals. Then, we could simply use this feature representation to train regressor models and predict BP by keeping BP values as labels. However, despite that this approach should prove a concise set of attributes of the PPG and ECG signals, this feature set may still not be suitable for BP prediction. On the other hand, from the literature, there have been many studies in which deep convolutional (CNN) networks were trained to extract features from PPG and/or ECG against BP labels and perform regression in the topmost layer to predict BP [24,25]. However, BP is used only as the label in these cases limiting the potential of utilizing the ABP waveform itself to predict BP. To utilize ABP features alongside PPG and ECG, we employed a different approach as follows. We trained the autoencoder with PPG and ECG signals as input and ABP waveform as the output anticipating that the network will inherently learn to map the ECG, PPG signals to the ABP waveforms. Consequently, through this process, the network is expected to map the various patterns of the PPG and ECG signals to the corresponding patterns in the ABP signal. Therefore, we hypothesize that by applying the aforementioned training mechanism of the autoencoder network, we can extract features from the PPG and ECG signals responsible for the changes in ABP. As a result, a regressor model, trained with these features, will likely be able to predict blood pressure better.

### 2.4. Pipeline for Blood Pressure (BP) Prediction

**Figure 4:**
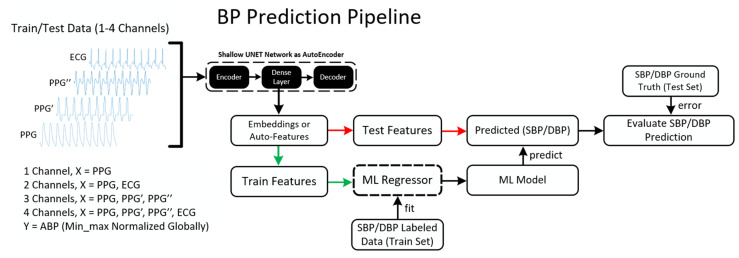
Complete pipeline for blood pressure (BP) prediction using a two-stage machine learning approach (dash means an iterative process).

The BP prediction pipeline consists of mainly two sections, namely the U-Net based autoencoder for feature extraction from the raw signals, and the machine learning-based regressor to perform regression on the extracted features for BP prediction. The complete pipeline is shown in Figure 4.

#### 2.4.1. Feature Extractor

The U-Net-based autoencoder is used for extracting a feature map from the raw input data. The dimensionality of the feature map may vary depending on the network setup (discussed elaborately in the experiments section). The general training setup for the U-Net based feature extractor (autoencoder) consisted of a batch size = 64, number of epochs = 100, patience (stopping criterion) = 15, mean squared error (MSE) as the loss function, Adam as the optimizer, and MAE as the metric being monitored. Batch size, number of training epochs, and the patience value were varied a few times initially to determine their optimal values.

#### 2.4.2. Regressor

Extracted features were regressed using traditional machine learning (ML) techniques, such as k-nearest neighbor (KNN), SVM, stochastic gradient descent (SGD), various ensemble techniques (e.g., adaptive boosting, gradient boosting, extreme gradient boosting (XGBoost 1.5.2, and random forest), and artificial neural network (ANN)-based MLP. For all these ML algorithms, various parameters were tweaked and tuned to get the optimum outcome. As shown in Supplementary Table S2, for MLP, Adam was chosen the solver, ReLU as the activation function, Invscaling as the learner, alpha = 0.0001, batch size = auto, max iteration = 500 and hidden layer size = 100. 

## 3. Experiments

The primary aim of the experiments was to find the best performing U-Net architecture which can be used as an autoencoder for optimum feature extraction. Later, the same pipeline can be used to evaluate external datasets. Therefore, mainly two types of experiments were performed in this study as discussed below.

### 3.1. Experiment 1 (Train and Test on UCI Dataset)

The UCI dataset (12,000 instances from 942 subjects) was originally divided into four equal ‘parts’. The first three parts of the UCI dataset were combined to make the train set (75% of the dataset) and the fourth part was taken as an independent test set (25% of the dataset). These four parts being independent in terms of subjects (i.e., no overlap of subject data across these parts). During training, a randomly selected 20% of the training set was used for validation. Four combinations of the four input signals, namely PPG, ECG, VPG, and APG were used in this experiment, while the target signal was ABP. Total predictor signal segments used for the four-channel approach (PPG, VPG, APG, and ECG as the four predictor signals) were 147,116 while the test set size was 53,043, as shown in Table 2.

Various sub-experiments were performed in Experiment 1 to determine the best U-Net architecture as an autoencoder (at least for this study). Their respective MAE was recorded in each case.

Variable depth of the encoder: the depth (number of levels) of the U-Net was varied from 1 to 4 to determine whether the depth of the architecture had any effect on the extracted latent features from the autoencoder.

Variable width of the encoder and number of features: the width of the encoder, which represents the number of kernels or filters present in the input layer, was varied from 32 to 256.

Variable kernel size: the kernel size was varied from 3 to 11 to see the effect of Kernel size on performance.

Variable number of channels: four combinations of the four predictor signals were used for BP prediction. For one channel: only PPG, for two channels: PPG and ECG, for three channels: PPG and its two derivatives, and for four channels: all four types of signal were utilized. 

Experiments on regression techniques: the extracted features were used to train some traditional Machine Learning regression techniques, namely, MLP, SGD, SVR, XGBoost, GradBoost, AdaBoost, k-nearest neighbor, and random forest to predict BP. 

BP Prediction from PPG-to-PPG Feature Mapping: Apart from the primary approach of this study which aimed at mapping PPG and ECG features to ABP features for BP prediction, an additional experiment was performed aiming at predicting BP by mapping PPG (or PPG and ECG) to PPG i.e., PPG was taken as the target signal instead of ABP while using the same BP labels and ground truths. The significance of this study lies in taking the ABP signal completely out of the equation which would help avoid acquiring simultaneous ABP data during data acquisition and BP can be predicted from PPG alone.

### 3.2. Experiment 2 (Validating on External “BCG Dataset”)

The external BCG dataset was investigated using two different methods. Firstly, the model trained on the whole UCI dataset is evaluated on the full BCG dataset (Method 1). Secondly, an exercise similar to Experiment 1 was performed on the UCI dataset, i.e., the model was trained using the BCG dataset through 5-Fold Cross-Validation (Method 2).

Train on UCI, test on BCG Dataset (Method 1): in this experiment, the BCG Dataset was tested against a model trained on the whole UCI Dataset. The outcome from this experiment proved the performance and generalizability of a model trained using the proposed shallow U-Net-based autoencoder on a completely unknown dataset. The training and testing sets used for this experiment are described in Table 3. 

Five-fold CV on BCG Dataset (Method 2): the BCG dataset was divided into train-test fold (80:20) and validated using a five-fold cross-validation approach. The training set, in this case, contained 1498 samples while the test set contained 374 samples (Table 4).

### 3.3. Evaluation Metrics

Primary Evaluation Metric:

Mean absolute error (MAE) [59] was used as the primary evaluation metric for this study. For example, for predicted values $\hat{y}$ = [*y*_1_, *y*_2_, *y*_3_, …, *y*_n_] and ground truth values *y* = [*y*_1_, *y*_2_, *y*_3_, …, *y*_n_], MAE is defined as in Equation (3) [57]:

(3) $$MAE=\frac{\sum_{i=1}^{n}\left|y_{i}-{\hat{y}}_{i}\right|}{n}$$

British Hypertension Society (BHS) Standard:

The BHS introduced a structured protocol [60] to act as a standard for assessing BP measuring devices and methods which has been frequently used in the literature as a metric. The BHS standard evaluates the performance based on absolute error while classifying the outcomes mainly into three categories, namely Grade A, B, and C. The grades are provided by measuring what percentage of the prediction absolute errors fall under (less than or equal to) 5 mmHg, 10 mmHg, and 15 mmHg, respectively. It is worth mentioning that for an algorithm or pipeline to obtain a certain grade, it has to satisfy the criteria of all three categories. There is also a Grade D for studies that fail to meet the requirements for Grade C [60].

Association for the Advancement of Medical Instrumentation (AAMI) Standard:

AAMI has proposed a similar standard [61] as BHS for evaluating BP measuring devices and algorithms. According to this standard, BP measuring systems should have a mean error (ME) and STD of magnitude less than or equal to 5 mmHg and 8 mmHg, respectively. Moreover, the number of subjects to be evaluated should be greater than or equal to 85.

Statistical analyses:

Mainly two types of statistical analysis were performed in this study, namely linear regression and the Bland–Altman plots [62]. The linear regression plots show the correlation between the ground truths and the predictions and can be represented by Equation (4) [63].

(4) $$Y_{i}=\beta_{0}+\beta_{1}X_{i}$$

Here, *Y_i_* and *X_i_* are the dependent and independent variables, respectively. *β*_0_ is the offset or the y-intercept and *β*_1_ is the slope. The most positive correlation results in a slope of 1, which in turn varies between −1 and 1. In this study, we also represent the linear correlation performance with the Pearson correlation coefficient (PCC). PCC is the covariance of the two variables divided by the product of their standard deviations, as shown in Equation (5) [63].

(5) $$r_{xy}=\frac{\sum_{i=1}^{n}\left(x_{i}-\bar{x}\right)\left(y_{i}-\bar{y}\right)}{\sqrt{\sum_{i=1}^{n}{\left(x_{i}-\bar{x}\right)}^{2}}\sqrt{\sum_{i=1}^{n}{\left(y_{i}-\bar{y}\right)}^{2}}}$$

Here, it is necessary to mention that the PCC formula for an entire population and a sample of the population is different due to considering population and sample means, respectively, during computation. In this case, PCC formulae for a sample have been used since the dataset is a sample of the originally collected dataset in MIMIC-II. On the contrary, we also computed and plotted the Bland–Altman plots to show the difference between the ground truths and the predictions over the whole BP range, which cannot be reflected upon properly from normal correlation plots.

## 4. Results

### 4.1. Experiment 1: Train and Test on UCI Dataset

Several different studies were carried out in Experiment 1 as mentioned earlier to identify the best network architecture with optimized parameters. In what follows, we will report the results of these studies. 

Variable depth of the encoder: as shown in Table 5, the MAE for BP prediction increased as the depth of the encoder increased. Based on this direct correlation, we can conclude that as the encoder became deeper, it increasingly looked into complex features of the signals and the network became lesser efficient in capturing peripheral features such as SBP and DBP. For this reason, the shallowest version of U-Net as an autoencoder model performed best for BP prediction.

**Figure 5:**
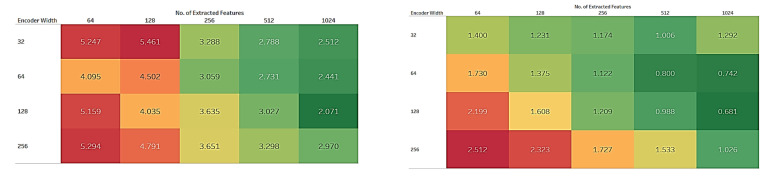
Heatmap depicting MAE for SBP (**left**) and DBP (**right**) prediction while varying encoder width and number of extracted features. Here, the color scale varies from red (high performance) to green (low performance).

Variable width of the encoder and number of features: as shown in Figure 5, the width of the input layer of the encoder varied from 32 to 256. The best performance was recorded at 128. The performance improved until 128 then started to drop again as the network becomes very wide and heavier than necessary. Here, the fixed parameters were encoder type, encoder depth, kernel size, number of channels, and regressor type.

The U-Net-based autoencoder was used to extract features from both train and test sets. So, the optimal number of features to be extracted is also crucial to investigate. Figure 5 also reveals that the performance gets better until 1024 features, then start dropping. There can be a misconception that more features will provide better accuracy indefinitely. But in this case, it was noticed that the performance does not increase, but rather drops slightly when the feature number is increased from 1024 to 2048 and the process becomes computationally expensive.

Variable number of channels: it can be noticed from Table 6 that performance improves by around 45% when two or three channels are used instead of using only PPG. The performance of the two and three-channel approaches are similar, while the performance improves again by around 25% when all four signals are used in combination. The same pattern was seen for both SBP and DBP even though SBP performed worse than DBP in all cases, which is a typical observation from the literature as well [12,13,14,15,16,18,20,21,22,23,24,25,26,27,64].

One significant outcome from this experiment is that PPG and its first two derivatives perform similarly to PPG alone with ECG for BP prediction. Therefore, ECG can be replaced just by deriving two derivatives of the PPG signal and supplying them as two additional channels in U-Net. Removing ECG while maintaining the performance greatly reduces the complexity of the test setup.

Variable kernel size: the kernel size, k = 3 performed best as the kernel size was varied from 3 to 11. The performance dropped as the kernel size was increased (Table 7).

**Figure 6:**
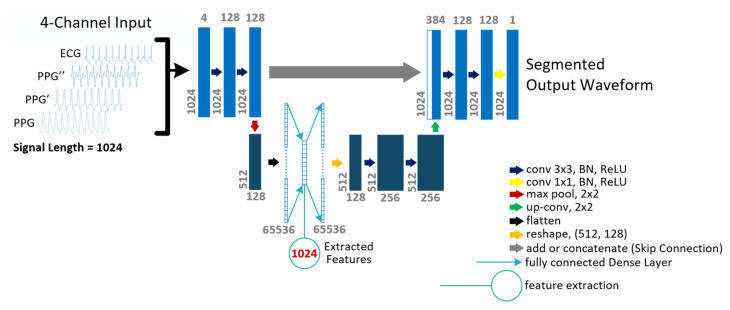
Architecture of the shallow U-Net model for feature extraction.

Based on these experiments, the best U-Net architecture as an autoencoder is shown in Figure 6 along with annotations for all parameters. Here, in the bottom layer of the U-Net, an extra fully connected dense layer was inserted to extract features. The number of parameters in the dense layer depends on the CNN block before it and the number of features to be extracted. For example, while extracting 1024 features, the size of the dense layer was (512 × 128 × 1024) = 67108864, which added up to the size of the whole model. It is worth mentioning that the dense layer could be placed between CNN blocks of 512 by 256 which would double the number of parameters (512 × 256 × 1024) but doing it did not improve the performance.

Experiments on regression techniques: the extracted features from the best autoencoder architecture, were trained using some traditional machine learning regression techniques to predict BP. As is evident from Table 8, MLP outperformed other classical machine learning techniques.

BP prediction from PPG-to-PPG feature mapping: from Supplementary Table S1, it can be seen that the PPG-to-PPG approach to predict BP was not very successful, at least using this pipeline, due to lower correspondence between BP values and PPG patterns. MAE for DBP and SBP prediction was around 7.7 and 17.1, respectively. This mini-experiment indirectly ascertained the robustness of the proposed pipeline in predicting BP by exploiting the relationship between BP values and corresponding ABP waveform patterns.

BHS Standard:

The criteria of the three grades along with the model performance of this study are presented in Table 9. From Table 9, it can be seen that with the developed pipeline, we have achieved Grade A for both SBP and DBP. For DBP prediction, in particular, almost 100% of the signals met the Grade A criterion.

Histograms of MAE for SBP and DBP predictions for all of them are provided in Supplementary Figure S2. It can be seen that for the DBP, the MAE for almost all predictions is below or equal to 5 mmHg, which is the Grade A threshold. On the other hand, for SBP, MAE of most of the predictions is below or equal to 5 mmHg, which is BHS Grade A, and MAE of almost all predictions is below or equal to 10 mmHg, which is BHS Grade B.

AAMI Standard:

As presented in Table 10, the predictions from our pipeline meet both categories of the AAMI standard keeping a large margin with the criteria.

Error measurements for all SBP and DBP predictions are plotted in Supplementary Figure S3. It can be seen that the error is normally distributed following the Central Limit Theorem. The SBP predictions are more widely distributed than the DBP predictions implying their higher deviation and lower accuracy.

**Figure 7:**
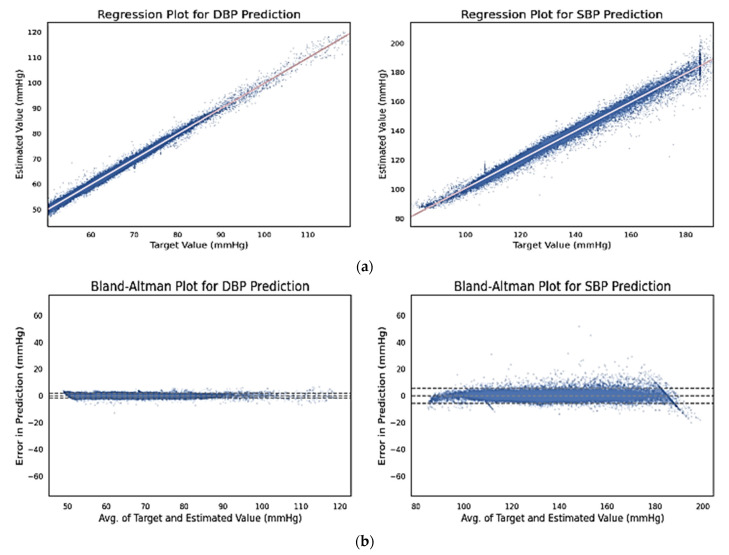
(**a**) Regression plots for DBP (**left**) and SBP (**right**) predictions vs. respective ground truths; (**b**) Bland–Altman plots for DBP (**left**) and SBP (**right**) predictions.

Statistical Analysis:

The response plots for SBP and DBP regression outcomes are shown in Figure 7a. From the plots, a high correlation between the target values and the ground truths is evident. The Pearson correlation coefficients for SBP and DBP predictions are 0.991 and 0.996, respectively, indicating a strong positive correlation between the target variables and the ground truths for both cases. On the other hand, *p*-values of approximately 0.01 for both cases indicate the statistical significance of the outcomes of this experiment when the test set contains 53,043 samples. Thus, the null hypothesis, which was rejected, stated that there is no relation between the predictions and the ground truths.

Figure 7b represents the Bland–Altman plots for DBP and SBP predictions, respectively. The 95% significance level, which is shown by the dashed lines, spans the segment from μ − 1.96σ to μ + 1.96σ, where μ and σ are population mean and standard deviation of the distribution, respectively. For SBP and DBP, the means are 5.618 and 1.933, respectively while the standard deviations are 2.89 and 0.894, respectively. Therefore, SBP and DBP spanned within the range [−0.046:11.282] and [0.181:3.685], respectively. It can be understood from Figure 7b that even though SBP samples deviated more (which is expected), in both cases most error terms fell within the dash marked 5 mmHg range. The presence of outliers is not severe, in fact very low for DBPs. Another important observation from the Bland–Altman plot is that the error magnitudes remain almost similar over the SBP and DBP ranges. Therefore, the error performances of ABP signals with extreme BP values (severe hypertension) were not affected by their high magnitude.

### 4.2. Experiment 2 (Validating on an External “BCG” Dataset)

In this experiment, after training on the whole pre-processed UCI dataset, the created model has been tested on the whole (similarly pre-processed) BCG dataset. The main aim was to prove the effectiveness of the shallow U-Net model trained on a large dataset on an external dataset.

Performance Evaluation:

MAE for SBP and DBP was found to be 2.728 and 1.166, respectively, after testing the whole BCG dataset with 1872 samples by the model trained on the whole UCI dataset. MAE was slightly higher than the results obtained from Experiment 1 with the UCI dataset but still better than any past study. The performance is excellent considering that the BCG dataset is completely unknown compared to the MIMIC-II (UCI repository) from all aspects of the data acquisition setup to data pre-processing. However, when there was no transfer learning, the MAE for five-Fold CV on the BCG dataset was found to be 6.336 and 2.658 for SBP and DBP, respectively. This can imply that the autoencoder requires a good amount of nicely varying balanced datasets to extract quality features. Therefore, it is important to train the proposed model using a large, general dataset that contains an ample number of features. Note that BHS and AAMI metrics information for external validation have not been provided since the number of patients in the BCG dataset does not suffice the minimum requirements for these metrics.

### 4.3. Comparison with Existing Works

Various research groups around the world attempted to predict BP from PPG and ECG signals separately or in combination using various machine learning techniques. It is hard to directly compare and evaluate the performances of those studies due to multiple factors such as the number of patients, data pre-processing, signal length, machine learning models, so on and so forth. In Table 11, only papers reporting their error performance in MAE have been reported. The entries in Table 11 are sorted in ascending order by the year of publication of the respective papers. Some works have low performance in terms of BHS and other metrics due to high standard deviation even though their error is low, which are reported in Table 12. 

Performance metrics such as MAE do not always show the complete picture of the performance of a study. For this reason, many studies in this domain represent their results in terms of BHS metrics. A comparison of BHS metrics of the current work with some past studies is shown in Table 12. As can be seen, only a handful of very recent studies could reach BHS Grade A for both SBP and DBP predictions. It is noticeable from Table 11 and Table 12 that even though some recent studies gained close or even better MAE than this study, they have lower performance in BHS metrics due to high deviation in the result (this can be further confirmed by comparing the respective AAMI metric). In terms of BHS, AAMI, and other metrics, our performance is the best so far, even with a larger dataset than almost all of these studies in terms of total signal duration. Moreover, the best performing shallow U-Net architecture proposed in this study as an autoencoder is also very lightweight. For example, the level-4, general version of U-Net used by Ibtehaz et al. [13] has approximately 10.5 million parameters without deep supervision (and it is just one of the two CNN networks used in the pipeline, the other one being the MultiResUNet [29]) while the shallow, level-1 U-Net model used for this experiment has only around 0.55 million parameters, around 19 times lighter.

### 4.4. Conclusions

This study aimed at developing a novel pipeline for BP prediction from PPG and ECG signals by experimenting with the U-Net architecture being used as an autoencoder to extract optimal features. Instead of the raw signals, the extracted features were regressed using machine learning techniques to predict SBP and DBP. The strength of this work lies in how the U-Net architecture was utilized for feature extraction, thereby achieving very high performance from the shallowest version of the U-Net architecture on the current largest possible dataset from the UCI repository. The extracted features were efficient enough in predicting the SBP and DBP, causing a significant performance boost compared to any previous study. Our lightweight network can be helpful for deployment in a resource-constrained setting. Independent test sets were used for evaluation purposes for both experiments performed in this study proving the robustness of the proposed pipeline. The dataset used for the second experiment was acquired through a completely different process (e.g., ABP was recorded non-invasively), but still our model achieved high performance when evaluated thereon thereby showing the generalizability thereof. This strongly suggests that extracting features from this large dataset using the shallow autoencoder provided the trained model with enough generalizable features to perform robustly even on external datasets. Some studies (e.g., [13]) reported that avoiding ECG signals as the second predictor, while maintaining high performance, could help in simplifying the hardware design, device implementation, and patient monitoring. The current study showed that even without the ECG signal, the model can perform similarly by just using the first two derivatives of PPG instead. MAE for SBP and DBP predictions with three channels were 2.74 and 0.96, respectively, which is still one of the best performances so far compared to the past studies. Therefore, a three-channel model (PPG and two derivatives) can easily be used for deployment without any ECG signal provided that the model is trained on a large general dataset (like the UCI dataset). One limitation in our approach can be the presence of motion artifacts or baseline wandering in the acquired signal for a mobile device such as wearables. Since the model was mostly trained on very clean signals collected in a clinical setup, it could greatly affect the model performance. While baseline wandering can be solved following many approaches (e.g., the one proposed in this paper) and motion artifact can also be corrected in many ways, one of which is proposed in this study for PPG signals [66]. Modern electronics have signal processing circuitry that can easily preprocess signals to get rid of this type of distortions before using them for BP prediction [67]. Moreover, for real-time, continuous BP monitoring, instead of the regressor, LSTM can be used instead according to these recent studies [37,40,41,42]. This approach will perform well given that the input features to the LSTM layers are optimal. To make the model robust enough to deal with data from various sources, it can be retrained with new data as a means of transfer learning. In conclusion, the proposed model and framework can be suitable for deployment in remote monitoring servers and mobile applications for real-time non-invasive BP monitoring applications.

## Figures and Tables

**Table 1:** Overview of the datasets (after pre-processing).

Datasets	BP Parameters	Minimum	Maximum	Mean	Standard Deviation
UCI Dataset	SBP	80.026	189.984	132.609	21.703
	DBP	50.000	119.927	63.705	9.978
	MAP	57.941	149.062	87.228	12.737
BCG Dataset	SBP	80.313	186.641	124.535	15.237
	DBP	43.899	96.829	65.011	9.180
	MAP	62.975	128.391	86.878	10.046

**Table 2:** Description of training and testing sets for Experiment 1.

No. of Channels	Channels	Target	Total Samples in the Train Set	Total Samples in the Test Set
1	PPG	ABP	147,116	53,043
2	PPG, ECG	ABP	147,116	53,043
3	PPG, VPG, APG	ABP	147,116	53,043
4	PPG, VPG, APG, ECG	ABP	147,116	53,043

**Table 3:** Description of training and testing sets for Experiment 2 (Method 1).

No. of Channels	Channels	Target	Total Samples in the Train Set from UCI	Total Samples in the Test Set from BCG
1	PPG	ABP	200,159	1872
2	PPG, ECG	ABP	200,159	1872
3	PPG, VPG, APG	ABP	200,159	1872
4	PPG, VPG, APG, ECG	ABP	200,159	1872

**Table 4:** Description of training and testing sets for Experiment 2 (Method 2).

No. of Channels	Channels	Target	Total Samples in the Train Set	Total Samples in the Test Set
1	PPG	ABP	1498	374
2	PPG, ECG	ABP	1498	374
3	PPG, VPG, APG	ABP	1498	374
4	PPG, VPG, APG, ECG	ABP	1498	374

**Table 5:** Mean absolute error (MAE) of BP prediction for variable encoder depth.

Fixed Parameters	Encoder Levels	MAE	
		SBP	DBP
Encoder Type: U-Net	1	2.333	0.713
Encoder Width: 128	2	3.169	1.099
Kernel Size: 3	3	3.763	1.243
No. of Channels: 4	4	4.416	1.419
No. of Extracted Feature: 1024			
Regressor: MLP			

**Table 6:** MAE of BP prediction for variable channels.

Fixed Parameters	No. of Channels	MAE	
		SBP	DBP
Encoder Type: U-Net	1	4.971	1.361
Encoder Depth: 1	2	2.513	0.825
Encoder Width: 128	3	2.739	0.960
Kernel Size: 3	4	2.333	0.713
No. of Extracted Feature: 1024			
Regressor: MLP			

**Table 7:** MAE of BP prediction for variable kernel or filter size.

Fixed Parameters	Kernel Size	MAE	
		SBP	DBP
Encoder Type: U-Net	1	2.387	0.876
Encoder Depth: 1	3	2.333	0.713
Encoder Width: 128	5	2.503	0.949
No. of Channels: 4	7	2.900	0.888
No. of Extracted Feature: 1024	9	3.421	1.568
Regressor: MLP	11	4.544	1.388

**Table 8:** MAE of systolic blood pressure (SBP) and diastolic blood pressure (DBP) for different machine learning (ML) techniques in Experiment 1.

Fixed Parameters	Regressor Algorithm	MAE for SBP	MAE for DBP
Encoder Type: U-Net Encoder Depth: 1 Encoder Width: 128 Kernel Size: 3 No. of Channels: 4 No. of Extracted Feature: 1024	MLP	2.333	0.713
	GradBoost	5.837	1.418
	SGD	5.945	2.261
	SVM	5.980	2.269
	XGBoost	6.089	1.429
	K-Nearest Neighbor	6.543	1.510
	AdaBoost	8.584	2.234

**Table 9:** Evaluation of BP prediction in Experiment 1 in terms of British Hypertension Society (BHS) Standard.

		Cumulative Error Percentage		
		≤5 mmHg	≤10 mmHg	≤15 mmHg
Our Results	SBP	92.02%	99.18%	99.85%
	DBP	99.01%	99.91%	100.0%
BHS Metric	Grade A	60%	85%	95%
	Grade B	50%	75%	90%
	Grade C	40%	65%	85%

**Table 10:** Evaluation of BP prediction in Experiment 1 in terms of Association for the Advancement of Medical Instrumentation (AAMI) standard.

		ME (mmHg)	STD (mmHg)	Number of Subjects
Our Results	SBP	0.09	0.94	942
	DBP	−0.019	2.876	
AAMI Standard		≤5 mmHg	≤8 mmHg	≥85

**Table 11:** Comparison of past studies based on MAE performance.

Study	Year Published	Dataset	Input Signals	Method	MAE (mmHg)	
					SBP	DBP
Kurylayak et al. [18]	2013	MIMIC, 15,000 Pulsations	PPG	ANN	3.80	2.21
Wang et al. [19]	2018	MIMIC, 72 Subjects	PPG	ANN	4.02	2.27
Slapničar et al. [24]	2019	MIMIC, 510 Subjects	PPG	CNN	9.43	6.88
Miao et al. [40]	2019	1711 ICU and 30 Arrythmia Patients	ECG	CNN + LSTM	7.10	4.61
Esmaelpoor et al. [39]	2020	MIMIC-II (200 Subjects)	PPG	CNN + LSTM	1.91	0.67
Ibtehaz et al. [13]	2020	MIMIC-II (942 Subjects)	PPG	CNN + CNN	5.73	3.45
Li et al. [22]	2020	MIMIC-II (3000 Records from UCI Repository)	PPG, ECG	LSTM	4.63	3.15
Hsu et al. [21]	2020	MIMIC-II (9000 Records from UCI Repository)	PPG, ECG	ANN	3.21	2.23
Athaya et al. [25]	2021	MIMIC-II (100 Subjects)	PPG	CNN	3.68	1.97
Harfiya et al. [23]	2021	MIMIC-II (5289 Records from UCI Repository)	PPG	LSTM	4.05	2.41
Baker et al. [65]	2021	MIMIC-III	PPG, ECG	CNN + LSTM	4.41	2.91
Rong et al. [66]	2021	MIMIC-II (UCI Repository)	PPG	CNN + LSTM	5.59	3.36
Sagirova et al. [64]	2021	512 Patients	ECG, PPG			
Qin et al. [41]	2021	MIMIC-II (1227 Records from UCI Repository)	PPG	VAE	7.95	4.11
This Study	2021	MIMIC-II (942 Subjects–12,000 Recordings from UCI Repository)	PPG, ECG	CNN + ANN	2.333	0.713
		MIMIC-II + BCG (942 + 40 = 982 Subjects)			2.728	1.166
AAMI Standard					≤5	

Note: It is important to mention that Hsu et al. [23] in their paper reported that they used 9000 subjects’ data for BP prediction from the UCI repository, but it was 9000 out of 12,000 instances or recordings of data collected from the MIMIC-II dataset. These are the data from 942 patients as reported by Kachuee et al. [14], the originator of this dataset. A similar occurrence happened for the case of Harfiya et al. [25] where they reported 5289 signal instances from the UCI repository as 5289 patients. In comparison, this study fully utilized all 12,000 instances.

**Table 12:** Comparison of past studies based on their performance of BHS metrics.

Study	SBP (%) in BHS Metrics				DBP (%) in BHS Metrics			
	Grade A	Grade B	Grade C	Attained Grade	Grade A	Grade B	Grade C	Attained Grade
Esmaelpoor et al. [39]	74	94	98	A	93	99	100	A
Ibtehaz et al. [13]	71	85	91	B	83	92	96	A
Li et al. [22]	60	80	89	B	77	96	100	A
Hsu et al. [21]	81	96	98	A	90	98	100	A
Athaya et al. [25]	76	94	99	A	94	99	100	A
Harfiya et al. [23]	71	94	99	A	91	99	100	A
Miao et al. [40]	50	76	90	B	66	90	97	A
Baker et al. [65]	68	90	97	A	83	96	99	A
Rong et al. [66]	54	87	94	B	83	95	98	A
Qin et al. [41]	59	86	95	B	82	96	99	A
This Study	92	99	99	A	99	~100	100	A

*%: The percentage of predicted signals falling within 5 (Grade A), 10 (Grade B), and 15 (Grade C) mmHg of their respective ground truth signals, respectively.

## Data Availability

The data used in this experiment along with other relevant documents used to complete this work have been provided in the following GitHub repository [68].

## Supplementary Materials

The following supporting information can be downloaded at: https://www.mdpi.com/article/10.3390/s22030919/s1, Figure S1: Some examples of bad signals automatically detected by the algorithm and removed.; Figure S2: Histogram of the MAE for SBP (left) and DBP (right) (a) and histogram of Mean Error (ME) for SBP (left) and DBP (right) (b). Table S1: PPG-to-PPG Performance for Variable Channels (1 to 4).
