# Supplementary material for: A Shallow U-Net Architecture for Reliably Predicting Blood Pressure (BP) from Photoplethysmogram (PPG) and Electrocardiogram (ECG) Signals
Source: Sensors (Basel). 2022 Jan 25;22(3):919. doi: 10.3390/s22030919 (PMC8840244; doi:10.3390/s22030919)
Supplement: Supplementary file 1 [file sensors-22-00919-s001.zip › sensors-1502120-supplementary.pdf]

Supplementary Materials

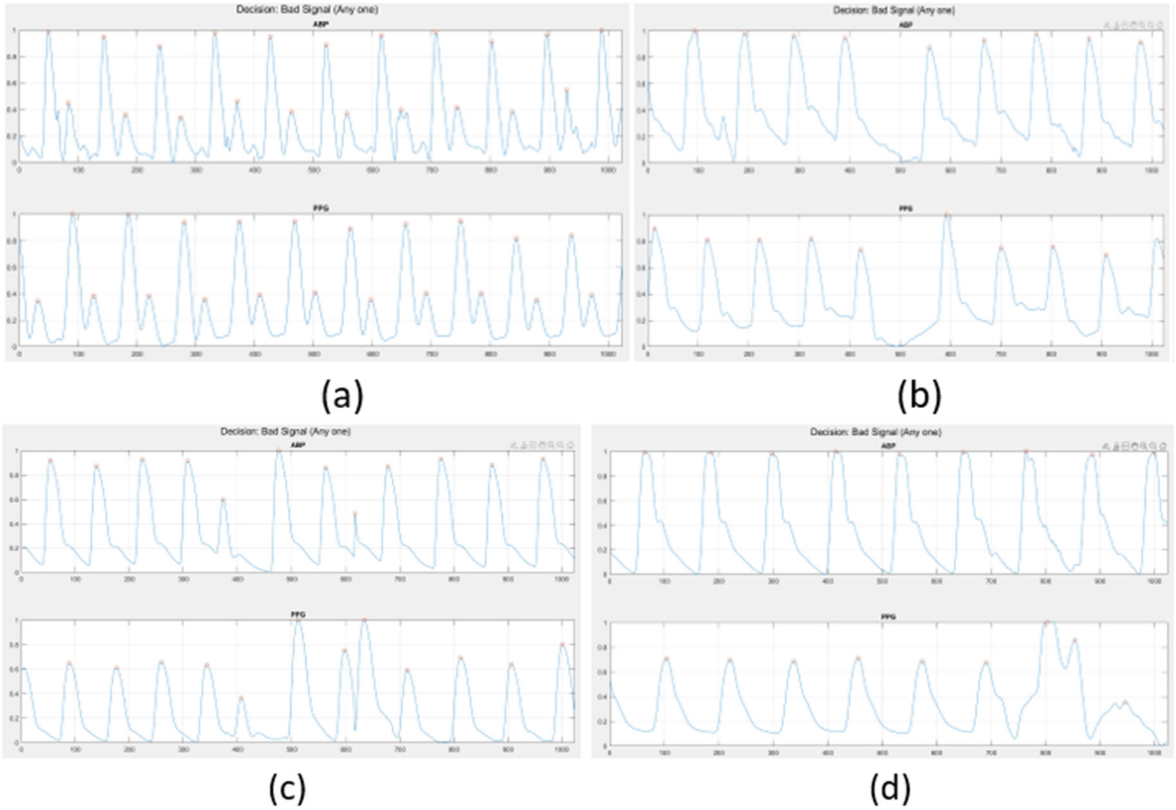

Figure S1: Some examples of bad signals automatically detected by the algorithm and removed.

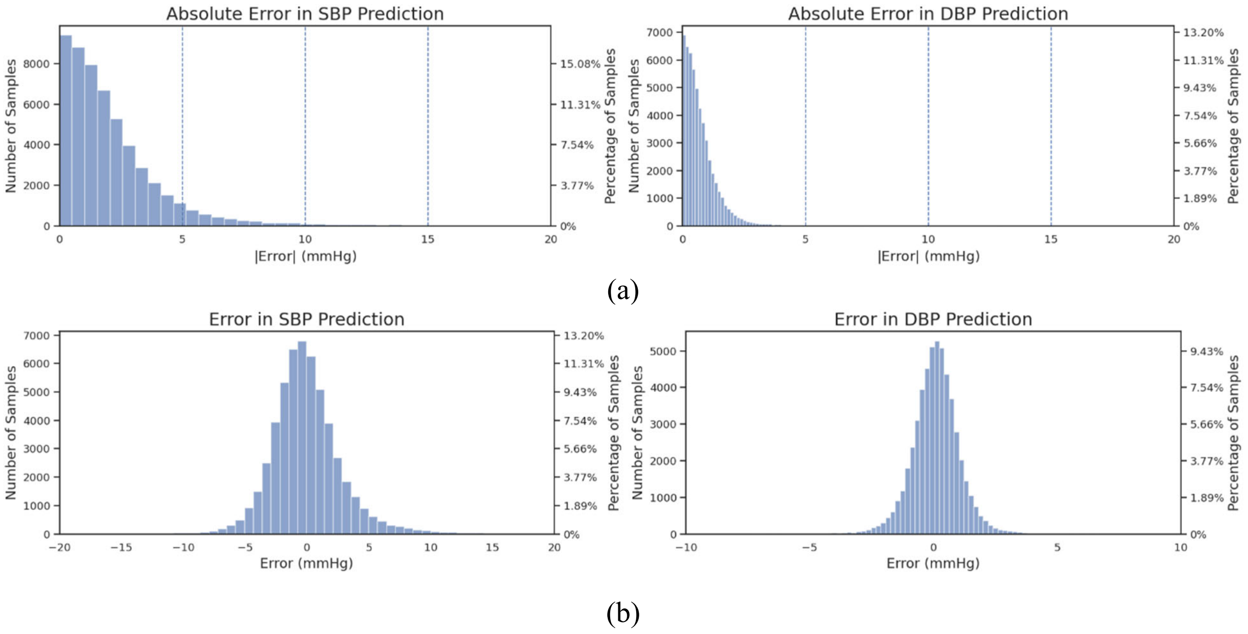

Figure S2: Histogram of the MAE for SBP (left) and DBP (right) (a) and histogram of Mean Error (ME) for SBP (left) and DBP (right) (b).

Table S1. PPG-to-PPG Performance for Variable Channels (1 to 4)

Encoder Type	Encoder Levels	Encoder Width	No. of Features	ML Algorithm	Channels	Kernel Size	MAE for SBP	MAE for DBP
**U-Net**	**1**	**128**	**1024**	**MLP**	1	**3**	17.084	7.679
					2		17.258	7.556
					3		17.358	7.720
					4		17.728	7.625
